# Psychometric properties of the FACT-M questionnaire in patients with Merkel cell carcinoma

**DOI:** 10.1186/s12955-017-0815-5

**Published:** 2017-12-22

**Authors:** Murtuza Bharmal, Fatoumata Fofana, Carla Dias Barbosa, Paul Williams, Lisa Mahnke, Alexia Marrel, Michael Schlichting

**Affiliations:** 1 Merck KGaA, Darmstadt, Germany; 2 Patient-Centered Outcomes, Mapi, Leiden, the Netherlands; 3 Patient-Centered Outcomes, Mapi, Lyon, France; 4 EMD Serono, Boston, MA USA

**Keywords:** Quality of life, Merkel cell carcinoma, FACT-M questionnaire, Psychometric validation, Patient-reported outcomes

## Abstract

**Background:**

No validated disease-specific questionnaires exist to capture health-related quality of life (HRQoL) in patients with Merkel cell carcinoma (MCC). The Functional Assessment of Cancer Therapy – Melanoma (FACT-M) is validated in patients with melanoma, which shares many similarities with MCC. This paper reports the psychometric properties of the FACT-M in the metastatic MCC population.

**Methods:**

Data were collected as part of a single-arm, open-label, multicenter trial involving patients with metastatic MCC who had failed at least one previous line of chemotherapy. FACT-M and EQ-5D were administered at baseline, Week 7, Week 13, and Week 25. An optional interview was administered at the same time points. MCC-specific FACT-M scores were derived following a combined quantitative and qualitative approach. Reliability and construct validity of original and additional MCC-specific FACT-M scores were assessed at baseline. Capacity to detect change in tumor size was assessed from baseline to Week 7. Minimally important differences (MIDs) were computed using distribution and anchor-based methods.

**Results:**

Baseline assessments were available in 70 patients (mean age: 70 years; 74.3% male); 19 patients were interviewed at baseline. Additional MCC-specific scores were as follows: Physical Function score (six items), Psychological Impact score (six items), and MCC summary score (12 items). FACT-M original and additional MCC-specific scores both demonstrated acceptable psychometric properties: high reliability (Cronbach’s alpha: 0.81–0.96), good convergent validity (correlations above 0.4 observed for 88% of items of the Melanoma surgery scale, 75% of items of the Melanoma scale, and 100% of items of the other FACT-M domains). Some evidence of floor/ceiling effects and poor discriminant ability was found. Higher scores (better HRQoL) on all FACT-M domains were observed in patients with better functioning (assessed by ECOG performance score), supporting clinical validity. Despite the small sample for responsiveness analysis (*n* = 37), the majority of FACT-M scores showed sensitivity to changes in tumor size at Week 7 with small to moderate effect sizes. MIDs were consistent with previously reported values in the literature for FACT-M domains.

**Conclusions:**

FACT-M is suitable to capture HRQoL in patients with metastatic MCC, thus making it a potential candidate for assessing HRQoL in MCC trials.

**Trial registration:**

This study is a post-hoc analysis conducted on data collected in Part A of the JAVELIN Merkel 200 trial. This trial was registered on 2 June 2014 with ClinicalTrials.gov as NCT02155647.

## Background

Merkel cell carcinoma (MCC) is a rare and aggressive skin cancer associated with Merkel cell polyomavirus, exposure to ultraviolet irradiation, immunosuppression, and old age [1, 2]. MCC occurs with an incidence of 0.2–0.4 cases per 100,000 people per year in Europe, 0.8 cases per 100,000 people per year in the United States of America, and 1.6 cases per 100,000 people per year in Australia [3–5]. The 5-year overall survival rate with metastatic MCC ranges from 0% to 18% based on retrospective analyses [6–9].

MCC is challenging to treat in metastatic stages due to limited treatment options and lack of standard therapeutic procedures. Avelumab is an anti-PD-L1 monoclonal antibody recently approved by the Food and Drug Administration (FDA) to treat patients 12 years and older with metastatic MCC (mMCC). Approval was based on data from an open-label, single-arm, multicenter clinical trial (JAVELIN Merkel 200 trial) including a cohort of patients who had previously progressed after chemotherapy for distant metastatic disease (Part A) as well as early data from patients naïve to systemic therapy in the metastatic setting (Part B) demonstrating a clinically meaningful and durable overall response rate [10–12].

The relevance of the assessment of how patients function and feel from their direct perspective is an important clinical endpoint in the literature. It has also been highlighted by both the FDA and the European Medicines Agency [13, 14]. Recently, the FDA emphasized the importance of patient-reported evaluation of disease-related symptoms, treatment-related symptoms, and physical functioning [15]. Questionnaires available to collect patient-reported outcomes (PROs) in patients with non-melanoma skin cancer and malignant melanoma were identified in a systematic literature review conducted in 2012 [16]. Nine cancer and skin cancer-specific PRO measures were identified for which adequate evidence of psychometric properties were available. Of these, the Functional Assessment of Cancer Therapy – General (FACT-G) and Functional Assessment of Cancer Therapy – Melanoma (FACT-M) provided evidence of acceptable psychometric properties. The FACT-G was only evaluated in patients with non-melanoma skin cancers. The FACT-M had more promising characteristics for patients with malignant melanomas, especially those with advanced disease, with good internal consistency of all scales, high reproducibility, and good sensitivity [17, 18]. In addition, significant correlations were reported between the FACT-M and other questionnaires measuring similar constructs (European Organisation for Research and Treatment of Cancer Quality of Life questionnaire [EORTC-QLQ] Melanoma; Profile of Mood States). Finally, the FACT-M was shown to distinguish between disease stages with significantly lower scores in patients with advanced (stages III or IV) melanoma than in patients with early-stage melanoma [19].

No validated disease-specific tools exist to capture health-related quality of life (HRQoL) in patients with MCC. The FDA encourages the use of adapted questionnaires in oncology, provided that the adaptation and validation follow a rigorous, scientific and sound approach [20]. As melanoma and MCC share many similarities, both being aggressive skin cancers, the FACT-M was considered as a potentially adequate tool to assess HRQoL in the MCC population. However, FACT-M psychometric properties have yet to be confirmed in patients with MCC.

This study was conducted to assess the reliability and validity of the FACT-M questionnaire in patients with mMCC.

## Methods

### Study design

A specific statistical analysis investigating psychometric properties of the FACT-M questionnaire in patients with mMCC was conducted on data collected in the avelumab clinical trial JAVELIN Merkel 200 (NCT02155647, [10]). This single-arm, open-label, multicenter trial was conducted in the United States of America, Europe, Australia, and Japan. Enrolled patients were male and female adults with histologically proven mMCC and an Eastern Cooperative Oncology Group performance status (ECOG PS) of 0 or 1 at trial entry, who had failed at least one line of chemotherapy. During the trial, patients received avelumab at a dose of 10 mg/kg as a 1-h intravenous infusion once every 2 weeks until significant clinical deterioration, unacceptable toxicity, or any criterion for withdrawal from the trial or trial drug was fulfilled. The primary analysis of the trial was performed in patients with a minimum of 6 months follow-up (date of data cutoff March 3, 2016), and the results have been published in Kaufman et al. (2016) [10, 11].

#### Data collected

HRQoL was assessed during the JAVELIN Merkel 200 trial using a generic questionnaire (EuroQol-5 Dimensions [EQ-5D), a melanoma-specific questionnaire (FACT-M), and optional subject qualitative interviews. FACT-M and EQ-5D were collected at sites using electronic tablets at baseline, throughout the treatment period (at Week 7 and then every 6 weeks) and at the End-of-Treatment visit. Optional qualitative patient interviews were conducted via telephone at baseline, Week 13, and Week 25.

Data used to assess the FACT-M psychometric properties were cut when all patients had at least 6 months of treatment follow up, and, as such, PRO data were available up to Week 25. Data collected during the baseline qualitative patient interviews were used to explore the content validity of the FACT-M in the MCC population.

#### FACT-M questionnaire

The FACT-M includes 51 items grouped into nine multi-item scores [17, 18]: six subscale scores and three summary scores. The six subscales consist of four subscales from the FACT-G (physical well-being [PWB], social well-being [SWB], emotional well-being [EWB], functional well-being [FWB]), one Melanoma scale, and one Melanoma surgery scale. The three summary scores include the FACT-M Trial Outcome Index (TOI), the FACT-G total score, and the FACT-M total score. The FACT-M administration guideline instructed patients to answer all items and select “Not at all” if they felt that the item was not applicable to them.

#### EQ-5D questionnaire

The EQ-5D is a self-administered, generic, utility questionnaire developed by the EuroQoL Group in 1990 [21]. It includes five single-item dimensions (mobility, self-care, usual activities, pain/discomfort, and anxiety/depression) and a vertical visual analogue scale (VAS) for the patients to rate their current health state. Patients must choose between five levels of difficulty in accomplishing tasks in each dimension (EQ-5D-5L). The responses to the five dimensions are used to create a utility index. In this study, utility values were calculated using country-specific value sets from the United States of America [22]. The VAS ranges from 0 (worst imaginable health state) to 100 (best imaginable health state).

#### Patient interviews

Interviews with patients were conducted to gather information regarding the impact of MCC and its treatments (radiotherapy or chemotherapy) on patients’ everyday lives, to assess patients’ experience of avelumab during the trial and to document the evolution of these experiences along the trial. Qualitative data obtained from these interviews were used to understand HRQoL concepts and symptoms in the MCC population, and these were compared to the concepts covered in the FACT-M in order to identify those most relevant items for mMCC patients.

### Statistical analyses

Statistical analyses were conducted on the PRO analysis set (PAS), which included all patients from the trial intent-to-treat population (i.e. all patients who received at least one dose of trial treatment) who completed at least one item of each PRO (FACT-M and EQ-5D) at baseline.

Identified concepts of interest in the MCC population included Physical Function, Visual Lesion Impact, and Psychological Impact. These concepts were identified following qualitative research in MCC and regulatory guidance.

MCC-specific FACT-M scores were derived by first selecting only those FACT-M items that matched to at least some extent concepts identified from the qualitative interviews, then by selecting FACT-M items that matched concepts of interest, and finally by conducting a statistical analysis assessing psychometric properties, including a data-reduction technique (principal component analysis).

Reliability and validity of scores for both original and additional MCC-specific FACT-M scores were assessed. Reliability is the degree to which an instrument is free from measurement error. Internal consistency reliability (the extent to which items within a domain are consistent with each other and measure a single underlying concept) was assessed at baseline using Cronbach’s alpha [23]. Validity is defined as the accuracy with which a measurement tool measures the concept it is intended to measure. Construct validity, i.e. confirmation of the scaling structure, clinical validity, and concurrent validity, were assessed at baseline. Scaling structure was confirmed using multitrait analysis assessing item convergent and discriminant validity. Clinical validity was assessed by comparing the FACT-M mean scale scores between groups of patients categorized by ECOG PS [24]; the hypothesis put forward being that patients with a better level of functioning should have better HRQoL. Concurrent validity was assessed at baseline by calculating the Pearson coefficient between the FACT-M scores and the EQ-5D VAS and Index score; the hypothesis was that domains measuring related concepts should have high correlation levels while domains measuring different concepts should have low correlations. Ability of the FACT-M scale to detect change over time was assessed from baseline to Week 7 by comparing change in FACT-M scale scores according to percentage change in tumor size (classified into three groups: reduction ≥30%, reduction between 0% and 30%, increase >0%) using paired t-test, effect size (ES), Standardized Response Mean (SRM), and Guyatt’s statistics [25, 26]. Tumor size reduction greater than 30% is consistent with RECIST 1.1 criteria for determining partial response, a commonly used clinical criteria in the evaluation of tumor burden [27]. Finally, minimally important differences (MIDs), defined as the smallest difference in score in the PRO domain that is perceived as meaningful and beneficial for the patient [28] were computed using distribution-based and anchor-based methods. Anchor-based MID thresholds were explored using the percentage change in tumor size at Week 7. The responder threshold was defined for each FACT-M score as the mean change from baseline to Week 7 in patients whose percentage change in tumor size decreased over 30%. The distribution-based method included the use of ES and standard error of measurement (SEM). Two responder thresholds were calculated as 0.2 x SD_BL_ and as 0.5 x SD_BL_, with SD_BL_ being the standard deviation of the score at baseline. The MID threshold using SEM was calculated as ${\mathrm{SD}}_{\mathrm{BL}}\mathrm{x}\sqrt{1-r}$ where r is the reliability coefficient. Recommended MID range for FACT-M scores in the mMCC population were identified based on maximum and minimum MID thresholds obtained using the different methods.

Comparison of quantitative variables between groups of patients was assessed using t-test when comparing two groups of patients or ANOVA when comparing three groups of patients or more. Statistical significance threshold was set to 5% for each two-sided test and is provided to aid interpretation. No adjustments were made to account for multiplicity. Statistical analyses were performed using SAS software for Windows (Version 9.4, SAS Institute Inc., Cary, NC, USA).

## Results

### Patient population

Among the 88 enrolled patients who received at least one dose of trial treatment, 70 patients completed at least one item of both EQ-5D and FACT-M questionnaires at baseline and were included in the PAS. The number of patients in the PAS was 49 at Week 7, 38 at Week 13, and 27 at Week 25. The optional interview was conducted with 19 patients.

Socio-demographics and clinical characteristics of patients in the PAS and interviewed patients are presented in Table 1 Overall, patients were mostly males (74.3%), with a mean age of 70.2 years. Patients were mainly from the USA (40 patients, 57.1%), followed by Europe (22 patients; 31.4%), and the rest of the world, i.e. Japan and Australia (8 patients; 11.4%). Socio-demographic and clinical characteristics of interviewed patients were very similar to those from the PAS, except that interviewed patients were mainly from the United States of America and none were from Asia.

**Table 1:** Patients’ socio-demographic and clinical characteristics at baseline

Characteristics^a^	PAS (*N* = 70)	Interviewed patients (*N* = 19)
Sex, n (%)		
Male	52 (74.3%)	15 (78.9%)
Female	18 (25.7%)	4 (21.1%)
Age, years		
Mean (SD)	70.2 (11.2)	72.2 (8.2)
Median	73.0	72.0
95% CI	67.5–72.9	68.3–76.2
Region, n (%)		
North America	40 (57.1%)	17 (89.5%)
Europe	22 (31.4%)	2 (10.5%)
Rest of the world	8 (11.4%)	0 (0.0%)
ECOG PS at baseline (n, %)		
0	38 (54.3%)	10 (52.6%)
1	32 (45.7%)	9 (47.4%)
Site of primary tumor (n, %)		
Non-skin	9 (12.9%)	4 (21.1%)
Skin	55 (78.6%)	14 (73.7%)
Missing	6 (8.6%)	1 (5.3%)
Tumor size at baseline (mm)		
n (missing)	61 (9)	16 (3)
Mean (SD)	103.7 (79.7)	83.3 (49.9)
Median	83.0	78.5
95% CI	83.3–124.1	56.7–109.8
Time since initial diagnosis (years)		
Mean (SD)	2.2 (0.8)	2.3 (0.8)
Median	2.0	2.0
95% CI	2.0–2.4	2.0–2.7
Time since first metastatic disease (months)		
Mean (SD)	16.9 (23.4)	16.3 (10.4)
Median	9.5	14.0
95% CI	11.4–22.5	11.3–21.2
Number of previous therapy lines (n, %)		
Mean (SD)	1.5 (0.7)	1.3 (0.5)
Median	1.0	1.0
95% CI	1.3–1.6	1.1–1.6

*CI* confidence interval, *ECOG PS* Eastern Cooperative Oncology Group performance status, *PAS* PRO analysis set, *SD* standard deviation

ECOG PS: 0 = fully active; 1 = restricted in physically strenuous activity

^a^Missing data included in calculation of percentages

### Development of the additional MCC-specific FACT-M scores

Additional MCC-specific FACT-M scores were developed to obtain constructs of interest specific to the mMCC population with specific focus items. Forty-three items from the FACT-M questionnaire were initially selected, corresponding to the core FACT-G and the Melanoma Subscale; the Melanoma surgery scale was not deemed relevant to the disease and objective of the trial. The iterative and combined qualitative and quantitative process allowed to derive three additional MCC-specific scores: Physical Function score (six items), Psychological Impact score (six items), and MCC summary score (sum of Physical Function and Psychological Impact scores; 12 items) (Table 2).

**Table 2:** Original and additional MCC-specific FACT-M scores

FACT-M Subscales and Summary scores	# of items	Items/Scales	Score range^a^
FACT-G subscales			
Physical well-being (PWB)	7	GP1 – GP7	0–28
Social/Family well-being (SWB)	7	GS1 – GS7	0–28
Emotional well-being (EWB)	6	GE1 – GE6	0–24
Functional well-being (FWB)	7	GF1 – GF7	0–28
Melanoma-specific subscales			
Melanoma Subscale (MS)	16	M1-M3, B1, ITU4, An10, Hep3, C1, C6, M5, M6, ITU3, MS8, M8, M9, HI7	0–64
Melanoma surgery Scale (MSS)	8	M10-M17	0–32
Summary score			
FACT-M TOI	30	PWB + FWB + MS	0–120
FACT-G total score	27	PWB + SWB + EWB + FWB	0–108
FACT-M total score	43	PWB + SWB + EWB + FWB + MS	0–172
MCC-specific scores			
Physical Function score (PF)		GP1, GP3, GF1, GF3, GF7, ITU4	0–24
Psychological Impact score (PI)		GE1, GE3 - GE6, MS8	0–24
MCC summary score		PF + PI	0–48

*MCC* Merkel cell carcinoma, *TOI* Trial Outcome Index

*GP* GP1- lack of energy; GP2- nausea; GP3- troubles meeting family needs; GP4- pain; GP5- side effects; GP6- feeling ill; GP7- lying in bed

*GS* GS1- close friendship; GS2- emotional support from family; GS3- support from friends; GS4- family accept illness; GS5- communication with family; GS6- close to partner; GS7- sex life

*GE* GE1- sadness; GE2- coping with illness; GE3- losing hope; GE4- nervousness; GE5- worry about death; GE6- worry about aggravation of disease

*GF* GF1- able to work; GF2- fulfilling work; GF3- enjoy life; GF4- illness acceptance; GF5- sleeping well; GF6- fun; GF7- quality of life

*M* M1- pain; M2- skin deterioration; M3- scars appearance; M5- pain in bones; M6- bloody stools; M8- remoteness; M9- cognitive functioning; M10- swelling (melanoma); M11- swelling (surgery); M12- swelling botherness; M13- painful movement; M14- swelling prevent activities; M15- swelling prevent nice clothing; M16- numbness; M17- range of movement

*B* B1- short breath

*ITU* ITU3- limited social activity; ITU4- limited physical activity

*AN* AN10- headaches

*Hep* Hep3- fevers

*C* C1- stomach swelling or cramp; C6- appetite

*MS* MS8- overwhelming condition

*HI* HI7- fatigue

^a^Higher score = better health-related quality of life

### Description of original FACT-M scale and additional MCC-specific scale scores over time

Original FACT-M scores and additional MCC-specific scores were rather steady over time, with a slight tendency to increase (better HRQoL) (Table 3).

**Table 3:** FACT-M scores and MCC-specific scores from baseline to each measurement time point

FACT-M Subscales and Summary scores	Score range^a^	Score - Mean (SD)				
		Baseline (*N* = 70)	Week 7 (*N* = 49)	Week 13 (*N* = 38)	Week 19 (*N* = 29)	Week 25 (*N* = 27)
FACT-G subscales						
Physical well-being (PWB)	0–28	21.8 (5.69)	21.3 (5.50)	22.5 (4.23)	22.3 (5.33)	23.4 (3.53)
Social/Family well-being (SWB)	0–28	22.4 (5.04)	21.5 (5.90)	21.2 (4.90)	22.7 (4.02)	23.1 (4.37)
Emotional well-being (EWB)	0–24	17.5 (4.56)	18.4 (4.46)	19.0 (3.58)	18.1 (4.29)	19.4 (3.46)
Functional well-being (FWB)	0–28	16.6 (6.37)	16.7 (6.15)	16.3 (5.30)	16.6 (6.33)	18.6 (5.50)
Melanoma-specific subscales						
Melanoma Subscale (MS)	0–64	50.5 (9.00)	50.3 (8.77)	51.5 (7.71)	51.6 (8.22)	53.9 (6.52)
Melanoma surgery Scale (MSS)	0–32	26.1 (6.10)	25.8 (7.22)	26.4 (5.87)	25.9 (6.83)	28.6 (3.42)
Summary scales						
FACT-M TOI	0–120	88.9 (19.28)	88.3 (18.23)	90.3 (15.21)	90.5 (18.28)	95.8 (14.05)
FACT-G total score	0–108	78.2 (17.71)	77.8 (16.15)	79.0 (12.89)	79.7 (16.03)	84.5 (12.89)
FACT-M total score	0–172	128.7 (25.24)	128.1 (22.88)	130.5 (18.96)	131.3 (23.28)	138.4 (17.95)
MCC-specific scores						
Physical Function score (PF)	0–24	15.2 (5.73)	14.1 (5.58)	14.8 (5.11)	14.9 (5.79)	17.1 (4.43)
Psychological Impact score (PI)	0–24	17.4 (4.75)	18.7 (4.81)	19.3 (3.86)	18.3 (4.33)	19.2 (3.34)
MCC summary score	0–48	32.6 (9.53)	32.8 (9.07)	34.2 (7.66)	33.2 (8.64)	36.3 (6.91)

*MID* minimally important difference, *PAS* PRO analysis set, *SD* standard deviation

^a^Higher score = better health-related quality of life

Scores were assessed in the PAS (*N* = 70)

### Psychometric validation in the mMCC population of both original and additional MCC-specific FACT-M scores

#### Internal consistency reliability

FACT-M original and additional MCC-specific scores demonstrated very good internal consistency, with Cronbach’s alpha coefficient varying from 0.81 to 0.96 for the original FACT-M scores and from 0.85 to 0.91 for the additional MCC-specific scores (Table 4).

**Table 4:** Internal consistency reliability and clinical and concurrent validity of the FACT-M and MCC-specific scores in the mMCC population

FACT-M Subscales and Summary scores	# of items	Cronbach’s alpha^a^	Scale structure		ECOG PS (mean [SD])			EQ-5D VAS	EQ-5D index
			Range of item-subscale correlations^b^	Convergent^c^/ Divergent^d^ validity (% of items)	0 (*N* = 39)	1 (*N* = 31)	*p*-value^e^	Correlation^b^	Correlation^b^
FACT-G subscales									
Physical well-being (PWB)	7	0.86	0.49–0.81	100/57	23.21 (5.02)	20.10 (6.08)	0.0221	0.44	0.75
Social/Family well-being (SWB)	7	0.81	0.41–0.86	100/71	23.03 (4.78)	21.49 (5.30)	0.2065	0.15	0.24
Emotional well-being (EWB)	6	0.83	0.53–0.75	100/67	17.97 (4.22)	16.87 (4.95)	0.3179	0.32	0.53
Functional well-being (FWB)	7	0.87	0.49–0.82	100/71	17.41 (6.68)	15.48 (5.90)	0.2113	0.29	0.63
Melanoma-specific subscales									
Melanoma Subscale (MS)	16	0.85	0.24–0.85	75/31	51.64 (8.93)	49.10 (9.02)	0.2427	0.39	0.75
Melanoma surgery Scale (MSS)	8	0.84	0.15–0.81	88/63	26.51 (6.19)	25.58 (6.03)	0.5290	0.16	0.51
Summary scales									
FACT-M TOI	30	0.94	–	–	92.26 (18.75)	84.68 (19.40)	0.1026	0.41	0.78
FACT-G total score	27	0.94	–	–	81.62 (17.25)	73.95 (17.62)	0.0713	0.37	0.67
FACT-M total score	43	0.96	–	–	133.26 (24.66)	123.04 (25.21)	0.0926	0.40	0.74
MCC-specific scores									
Physical Function score (PF)	6	0.88	–	–	16.38 (5.67)	13.71 (5.54)	0.0519	0.37	0.72
Psychological Impact score (PI)	6	0.85	–	–	17.82 (4.40)	16.87 (5.18)	0.4101	0.30	0.52
MCC summary score	12	0.91	–	–	34.21 (8.99)	30.58 (9.94)	0.1145	0.37	0.69

*#* number, *ECOG PS* Eastern Cooperative Oncology Group performance status, *MCC* Merkel cell carcinoma, *PAS* PRO analysis set, *SD* standard deviation, *TOI* Trial Outcome Index

^a^Recommended threshold α > 0.7; ^b^Pearson correlation coefficients; ^c^% of items correlated with its own dimension ≥0.4; ^d^% of items correlated with its own dimension higher than the correlation with any other dimension; ^e^ *p*-value from t-test of score between the two ECOG PS groups at baseline

Internal consistency reliability and clinical validity were assessed in the PAS (*N* = 70)

ECOG PS: 0 = fully active; 1 = restricted in physically strenuous activity

#### Construct validity

Convergent validity criterion, i.e. Pearson’s correlation above 0.4, was met for 75% of the items of the Melanoma Subscale, 88% of the items of the Melanoma surgery scale, and 100% of the items of the 4 subscales of the FACT-G scale (PWB, SWB, EWB, and FWB) (Table 4). Discriminant validity criterion, met when correlation of an item with its own dimension is higher than the correlation of this item with all other dimensions, was met for 57% to 71% of the items of the subscales PWB, SWB, EWB, FWB, and Melanoma surgery scale but only 31% of the items in the Melanoma Subscale, suggesting poor discriminant validity of this subscale. Both original and additional MCC-specific FACT-M scores demonstrated clinical validity, with higher scores (better HRQoL) observed in patients with better functioning (assessed by ECOG PS) (Table 4). A significant difference was even observed for the PWB subscale (*p* = 0.0221). Similarly, a lower score in all domains of the FACT-M, except the Melanoma Subscale and the Melanoma surgery scale, was observed among patients with visceral metastases at baseline compared to those without visceral metastases (*p* > 0.05). The FACT-M questionnaire presented good concurrent validity, as high correlation coefficients were observed between the FACT-M scores and EQ-5D for items that represented the same underlying concept, and lower coefficients were observed when different concepts were assessed by the two instruments. In particular, higher coefficients were found between the EQ-5D index score and the FACT-M physical well-being dimension score and the Melanoma Subscale score (Table 5).

**Table 5:** Ability of the FACT-M to detect change and MIDs in the mMCC population

FACT-M Subscales and Summary scores	Comparison of change in score from baseline to Week 7 Percent change in tumor size (mean change in score [ES^[a]^])				MIDs assessed at Week 7				Published MIDs in melanoma population
	Change in tumor size				0.2 x SD_BL_	0.5 x SD_BL_	SEM^[d]^	Range(min-max)	
	Reduction ≥ 30%^[b]^ (*N* = 14)	Reduction between 0-30% (*N* = 7)	Increase >0% (*N* = 16)	*p*-value^[c]^					
FACT-G subscales									
Physical well-being (PWB)	0.21 (0.05)	-1.00 (-0.18)	-1.75 (-0.31)	0.631	1.14	2.85	2.15	0.21 - 2.85	2.00-3.00^[e]^
Social/Family well-being (SWB)	-0.49 (-0.10)	-0.45 (-0.12)	-1.74 (-0.28)	0.624	1.01	2.52	2.21	-0.49 - 2.52	-
Emotional well-being (EWB)	1.07 (0.22)	1.29 (0.20)	-0.25 (-0.09)	0.596	0.91	2.28	1.87	0.91 - 2.28	2.00^[e]^
Functional well-being (FWB)	2.64 (0.42)	-0.14 (-0.02)	-2.81 (-0.42)	0.005	1.27	3.19	2.26	1.27 - 3.19	2.00-3.00^[e]^
Melanoma-specific subscales									
Melanoma Subscale (MS)	2.71 (0.26)	-2.43 (-0.36)	-2.94 (-0.42)	0.051	1.80	4.50	3.46	1.80 - 4.50	2.00-4.00^[f]^
Melanoma surgery scale (MSS)	2.07 (0.34)	-3.00 (-1.02)	-3.13 (-0.49)	0.036	1.22	3.05	2.46	1.22 - 3.05	1.00-2.00^[f]^
Summary scales									
FACT-M TOI	5.57 (0.31)	-3.57 (-0.21)	-7.50 (-0.42)	0.038	3.86	9.64	4.84	3.86 - 9.64	5.00-9.00^[f]^
FACT-G total score	3.44 (0.21)	-0.31 (-0.02)	-6.55 (-0.35)	0.155	3.54	8.85	4.24	3.44 - 8.85	3.00-7.00^[e]^
FACT-M total score	6.15 (0.26)	-2.74 (-0.12)	-9.49 (-0.38)	0.074	5.05	12.62	5.26	5.05 - 12.62	-
MCC-Specific scores									
Physical Function score (PF)	0.71 (0.14)	-1.86 (-0.33)	-2.13 (-0.34)	0.153	1.15	2.87	1.88	0.71 - 2.87	-
Psychological Impact score (PI)	0.79 (0.15)	2.29 (0.39)	0.31 (0.10)	0.609	0.95	2.38	1.98	0.79 - 2.38	-
MCC summary score	1.50 (0.17)	0.43 (0.04)	-1.81 (-0.21)	0.530	1.91	4.76	2.91	1.50 - 4.76	-

*ES* effect size, *MCC* Merkel cell carcinoma, *MID* Minimally important difference, *PAS* PRO analysis set, *SD*_BL_ Standard deviation of the score at baseline, *SEM* Standard error of measurement, *TOI* Trial Outcome Index

Ability to detect change and MIDs of the FACT-M were assessed in the PAS (*N* = 70).

^[a]^ES around 0.20: small change; ES around 0.50: moderate change; ES around 0.80: large change; ^[b]^Reduction in tumor size ≥30% was used as the anchor for MID thresholds; ^[c]^ *p*-value from ANOVA comparing score change from baseline to week 7 between the three groups based on change in tumor size ^[d]^SEM was calculated using Cronbach's alpha for internal consistency reliability; ^[e]^Askew et al. 2009; ^[f]^Cella et al 2002

#### Ability to detect change and MID

Measurable tumor shrinkage greater than 30% (i.e. indicating partial/complete response) was associated with an improvement in HRQoL scores, whereas tumor growth was associated with a decrease in HRQoL scores. The magnitude of these change scores were in the region of small to moderate effect sizes for both tumor shrinkage and tumor increase groups (Table 5). Clear differentiation of change scores between groups was observed for FWB (*p* = 0.005), Melanoma surgery scale (*p* = 0.036), and TOI (*p* = 0.038). A similar trend was observed for the other scales and subscales with the exception of EWB, where a decrease was observed for the tumor shrinkage group.

Anchor-based calculations of the MIDs were generally consistent with distribution based calculations; MID ranges (min – max) based on the different calculation methods was generally in the range of 0.2 x SD_BL_ (lowest) to 0.5 x SD_BL_ (highest) (Table 5). Percentage of responders varied from 18% to 39% when response was defined as a change in score above SEM calculated MIDs and from 22% to 53% when response was defined as a change in score above anchor calculated MIDs (Table 6).

**Table 6:** Responder analysis based on various MID methods

FACT-M Subscales and Summary scores		Percentage of responders^[a]^ at Week 7 by MID calculation method		
	SEM^[b]^	Anchor^[c]^	Minimum threshold	Maximum threshold
FACT-G subscales				
Physical well-being (PWB)	18.4	32.7	32.7	18.4
Social/Family well-being (SWB)	22.4	53.1	53.1	18.4
Emotional well-being (EWB)	34.7	34.7	44.9	26.5
Functional well-being (FWB)	30.6	30.6	34.7	22.4
Melanoma-specific subscales				
Melanoma Subscale (MS)	22.4	26.5	34.7	20.4
Melanoma surgery scale (MSS)	22.4	22.4	30.6	20.4
Summary scales				
FACT-M TOI	24.5	22.4	30.6	14.3
FACT-G total score	30.6	34.7	34.7	20.4
FACT-M total score	28.6	28.6	28.6	14.3
MCC-Specific scores				
Physical Function score (PF)	18.4	26.5	26.5	12.2
Psychological Impact score (PI)	38.8	51.0	51.0	26.5
MCC summary score	24.5	30.6	30.6	18.4

*MCC* Merkel cell carcinoma, *MID* Minimally important difference, *PAS* PRO analysis set, *SEM* Standard error of measurement, *TOI* Trial Outcome Index

Percentage of responders were assessed in the PAS (*N* = 70)

^[a]^A responder is defined as a patient whose score had changed relative to baseline by an amount greater than or equal to the MID; ^[b]^SEM was calculated using Cronbach's alpha for internal consistency reliability; ^[c]^Reduction in tumor size ≥30% was used as the anchor for MID thresholds

## Discussion

The FACT-M is a validated questionnaire to assess HRQoL in patients with melanoma, a disease sharing similarities with MCC. As there is currently no existing validated disease-specific PRO questionnaire to capture HRQoL in patients with MCC, the FACT-M questionnaire was a candidate questionnaire to capture disease-specific HRQoL outcomes in mMCC.

Overall, psychometric properties of the FACT-M questionnaire in the mMCC population was acceptable, as the questionnaire demonstrated good item convergent validity, very good internal consistency reliability, clinical validity, and notable ability to detect change in tumor size, given the small sample size. Very good internal consistency ensured that items within a domain reflect a single underlying concept and responses to these items are consistent with each other. However, the FACT-M questionnaire demonstrated insufficient discriminant validity, especially for items of the Melanoma Subscale. A similar finding has been previously reported in the original FACT-M validation work [17] and is likely due to the addition disease-specific module items that correlate highly with core items but not necessarily together. Therefore, results from the Melanoma Subscale should be interpreted cautiously not only in patients with melanoma but also in patients with MCC. Altogether, these results showed a good construct validity of both original and additional MCC-specific FACT-M scores.

In an attempt to create scores with better psychometric properties for the mMCC population, additional scores were derived from concepts arising from patient interviews. In contrary to our expectation, these additional scores did not perform markedly better than the original FACT-M scores, suggesting that the original scores are sufficient for future studies in the mMCC population.

Trends in change scores were observed in both tumor shrinkage and tumor increase groups. MID values were consistent in magnitude with previously reported values in the literature for FACT-M domains [29, 30] as expected considering the similarities between the two diseases. The magnitude of these change scores were consistent with distribution based methods of MID calculation, which supports the choice of anchor for detecting differences in HRQoL domains. One unexplained finding was the small reduction in social/family well-being in the tumor shrinkage group, which requires further investigation. Ranges of MIDs for future studies involving the FACT-M in the mMCC population exclude this observed negative association and are as follows: PWB (1–3), SWB (1–3), EWB (1–3), FWB (2–4), MS (2–5), MSS (2–3), TOI (4–9), FACT-G total (4–8), and FACT-M total (5–12).

### Limitations and future directions

Data used in this study were collected in a clinical trial setting, which may have biased the study results: e.g. restrictive patient eligibility criteria may result in a study population not entirely representative of the mMCC population. Another limitation to the study results may be the small sample sizes. However, adequate quantity of analyzable data was retrieved from the PRO administered during the trial. This was likely due to protocol specific procedures such as training and reminders, electronic administration of PRO data (missing items were not permitted by the electronic questionnaires), and instructions provided by the FACT-M questionnaire. Indeed, patients were required to answer all items and select “Not at all” if they felt that the item is not applicable to them.

Further work could include performing specific cognitive debriefing interviews (i.e. an in-depth item per item review of the questionnaire by patients) that could provide additional insights on the relevance of the item selected for the study-specific scores.

## Conclusion

In conclusion, assessment of FACT-M psychometric properties demonstrated that FACT-M is suitable to capture HRQoL in patients with mMCC, thus making it a potential candidate for assessing HRQoL in mMCC trials. HRQoL improvements were observed in patients with relevant tumor shrinkage after 7 weeks of avelumab treatment for the majority of FACT-M scores, except the Social/Family well-being domain. This link between HRQoL and clinically relevant endpoints will be valuable to assess benefits of novel treatments for mMCC. Furthermore, as psychometric properties of the additional MCC-specific FACT-M scores were similar to the original ones with no evidence of superior measurement properties, the original FACT-M scores are the ones recommended to capture HRQoL in patients with mMCC.

## Abbreviations

ECOG PS: Eastern Cooperative Oncology Group performance status
EORTC-QLQ: European Organisation for Research and Treatment of Cancer Quality of Life questionnaire
EQ-5D: EuroQol-5 Dimensions
ES: Effect size
EWB: Emotional well-being
FACT-M: Functional Assessment of Cancer Therapy – Melanoma
FDA: Food and Drug Administration
FWB: Functional well-being
HRQoL: Health-related quality of life
MCC: Merkel cell carcinoma
MID: Minimally important differences
PF: Physical Function
PI: Psychological Impact
PRO: Patient-reported outcomes
PWB: Physical well-being
SD_BL_: Standard deviation of the score at baseline
SEM: Standard error of measurement
SRM: Standardized Response Mean
SWB: Social well-being
TOI: Trial Outcome Index
