# Correction to: Psychometric properties of the FACT-M questionnaire in patients with Merkel cell carcinoma

**DOI:** 10.1186/s12955-019-1122-0

**Published:** 2019-03-27

**Authors:** Murtuza Bharmal, Fatoumata Fofana, Carla Dias Barbosa, Paul Williams, Lisa Mahnke, Alexia Marrel, Michael Schlichting

**Affiliations:** 1 Merck KGaA, Darmstadt, Germany; 2 Patient-Centered Outcomes, Mapi, Leiden, the Netherlands; 3 Patient-Centered Outcomes, Mapi, Lyon, France; 4 EMD Serono, Boston, MA USA

**Correction to: Health and Quality of Life Outcomes (2017) 15:247**

**https://doi.org/10.1186/s12955-017-0815-5**

Unfortunately, the original version of this article [1] contained one error around the EQ-5D visual analogue scale (VAS) scores affecting a few patients.

In Table 4 (Table 1 here), the column presenting the correlations between the FACT-Melanoma (FACT-M) and Merkel cell carcinoma (MCC)-specific scores and the EQ-5D VAS scores have been revised. The corrected table is shown below. None of the interpretation of results or conclusions is affected.

**Table 1:** Internal consistency reliability and clinical and concurrent validity of the FACT-M and MCC-specific scores in the mMCC population

FACT-M Subscales and Summary scores	# items	Cronbach’s alpha^[a]^	Scale structure		ECOG PS (mean [SD])			EQ-5D VAS	EQ-5D index
			Range of item-subscale correlations^[b]^	Convergent^[c]^/Divergent^[d]^ validity (% of items)	0 (*n* = 39)	1 (*N* = 31)	*p*-value^[e]^	Correlation^[b]^	Correlation ^[b]^
FACT-G subscales									
Physical well-being (PWB)	7	0.86	0.49–0.81	100/ 57	23.21 (5.02)	20.10 (6.08)	0.0221	0.59	0.75
Social/Family well-being (SWB)	7	0.81	0.41–0.86	100/ 71	23.03 (4.78)	21.49 (5.30)	0.2065	0.19	0.24
Emotional well-being (EWB)	6	0.83	0.53–0.75	100/ 67	17.97 (4.22)	16.87 (4.95)	0.3179	0.36	0.53
Functional well-being (FWB)	7	0.87	0.49–0.82	100/ 71	17.41 (6.68)	15.48 (5.90)	0.2113	0.44	0.63
Melanoma-specific subscales									
Melanoma Subscale (MS)	16	0.85	0.24–0.85	75 / 31	51.64 (8.93)	49.10 (9.02)	0.2427	0.55	0.75
Melanoma surgery Scale (MSS)	8	0.84	0.15–0.81	88 / 63	26.51 (6.19)	25.58 (6.03)	0.5290	0.26	0.51
Summary scales									
FACT-M TOI	30	0.94	–	–	92.26 (18.75)	84.68 (19.40)	0.1026	0.58	0.78
FACT-G total score	27	0.94	–	–	81.62 (17.25)	73.95 (17.62)	0.0713	0.50	0.67
FACT-M total score	43	0.96	–	–	133.26 (24.66)	123.04 (25.21)	0.0926	0.55	0.74
MCC-Specific scores									
Physical function score (PF)	6	0.88	–	–	16.38 (5.67)	13.71 (5.54)	0.0519	0.52	0.72
Psychological impact score (PI)	6	0.85	–	–	17.82 (4.40)	16.87 (5.18)	0.4101	0.36	0.52
MCC summary score	12	0.91	–	–	34.21 (8.99)	30.58 (9.94)	0.1145	0.49	0.69

# number, *ECOG PS* Eastern Cooperative Oncology Group performance score, *MCC* Merkel cell carcinoma, *PAS* PRO analysis set, *SD* Standard deviation, *TOI* Trial Outcome Index

[a] Recommended threshold α > 0.7; [b] Pearson correlation coefficients; [c] % of items correlated with its own dimension ≥0.4; [d] % of items correlated with its own dimension higher that the correlation with any other dimension; [e] *p*-value from t-test of score between the two ECOG performance status groups at baseline

Internal consistency reliability and clinical validity were assessed in the PAS (*N* = 70)

ECOG PS: 0 = Fully active; 1 = Restricted in physically strenuous activity
